# Evaluation of the cell culture based and the mouse brain derived inactivated vaccines against Crimean-Congo hemorrhagic fever virus in transiently immune-suppressed (IS) mouse model

**DOI:** 10.1371/journal.pntd.0008834

**Published:** 2020-11-23

**Authors:** Shaikh Terkis Islam Pavel, Hazel Yetiskin, Ahmet Kalkan, Aykut Ozdarendeli

**Affiliations:** 1 Department of Microbiology, Medical Faculty, Erciyes University, Kayseri, Turkey; 2 Vaccine Research, Development and Application Center, Erciyes University, Kayseri, Turkey; 3 Department of Infectious Diseases and Clinical Microbiology, Medical Faculty, Karadeniz Technical University, Trabzon, Turkey; University of Texas Medical Branch, UNITED STATES

## Abstract

Crimean-Congo hemorrhagic fever virus (CCHFV) is a tick-borne virus in the *Nairoviridae* family within the *Bunyavirales* order of viruses. Crimean-Congo hemorrhagic fever (CCHF) is the most widespread among tick-borne human viral diseases. It is endemic in many areas of Africa, Asia, the Middle East, in the Balkans, Russia and countries of the former Soviet Union. The confirmed CCHF cases were seen in Spain in 2016 to signify expansion of the virus into new geographical areas. CCHFV causes a viral human disease characterized by sudden onset of fever, headache, abdominal pain, nausea, hypotension, hemorrhage, and hepatic dysfunction with fatality rates up to 30%. Currently, there are no spesific treatments or licensed vaccines available for CCHFV. The absence of a susceptible animal model for CCHFV infection was severely hindered work on the development of vaccines. However, several animal models of CCHFV infection have been recently developed and used to assess vaccine efficacy. In this study, we have used the transiently immune-suppressed (IS) mouse model that MAb-5A3 was used to block IFN-I signaling in immune intact, wild-type mice at the time of CCHFV infection to evaluate the immune response and efficacy of the cell culture based and the mouse brain derived inactivated vaccines against CCHFV. Both vaccine preparations have provided complete protection but the cell culture based vaccine more effectively induced to CCFHV spesific antibodies and T cell responses. This is the first comparison of the cell culture based and the mouse brain derived vaccines for assessing the protective efficacy and the immunogenicity in the IS mouse CCHFV model.

## Author summary

Crimean-Congo hemorrhagic fever is one of the most medically important tick-borne disease. CCHFV causes severe clinical signs in humans but not in its vertebrate animal hosts. Transmission of the virus to humans could be through the bite of infected ticks or by exposure to the tissues or blood of infected animals. Nosocomial infections have also been reported in endemic countries and are considered to be significant problems for health-care workers and family members. With the increasing number of the cases and expanding new foci of the CCHF endemic area, there is an urgent need to prioritize control strategies on the consideration of disease reduction. Recently, different approaches have been attempted towards the development of a CCHFV vaccine. We previously developed a cell culture based inactivated CCHFV vaccine that demonstrated protective efficacy in IFNAR^-/-^ mice. To date, the only CCHFV vaccine tested in humans is the suckling mouse brain derived vaccine used only in Bulgaria. However, there are no experimental data to show whether this vaccine confers to protective efficacy in a mouse model. We have used the IS mouse model to explore the immunogenicity and vaccine efficacy of the cell culture based and the mouse brain derived inactivated vaccines against CCHFV. Both vaccine preparations have provided complete protection after challenge studies but the cell culture based vaccine more effectively induced to CCFHV spesific antibody and T cell responses.

## Introduction

Crimean-Congo hemorrhagic fever (CCHF) is the most medically important tick-borne disease [1,2]. The causative agent is Crimean-Congo hemorrhagic fever virus (CCHFV) is a member of the genus *Orthonairovirus* and family *Nairoviridae* in the *Bunyavirales* order [1–3]. The virus is maintained in nature in an enzootic tick-vertebrate-tick cycle [3–5]. Although the CCHFV has been found in thirty different species of ticks the main vector and a reservoir of CCHFV are *Hyalomma* ticks [3,5]. The CCHFV is transmitted horizontally (transstadial, venereal transmission, and co-feeding,) and vertically (transovarial) within the tick population [3,5–7]. *Hyalomma* ticks feed on numerous domestic and wild animals serve as amplifying hosts for the virus [2,3,7]. The disease is endemic in wide areas of Africa, Asia, Eastern Europe and the Middle East which is considered for geographic distribution of *Hyalomma* ticks [1,3,4]. During the last two decades, new endemic areas of CCHFV have have been reported in the Balkan Peninsula, southwest Russia, the Middle East, western China, India, Africa, Turkey and Spain [3,4,8].

CCHFV causes severe clinical signs in humans but not in its vertebrate animal hosts [1–3]. Transmission of the virus to humans could be through the bite of infected ticks or by exposure to the tissues or blood of infected animals [3,5,6]. Nosocomial infections have also been reported in endemic countries and are considered to be significant problems for health-care workers and family members [1–3]. After an incubation period of 1–13 days, the onset of disease is sudden, with flu-like symptoms such as fever, dizziness, myalgia, headache, nausea, vomiting and neck pain. In severe cases, the hemorrhagic period develops rapidly, usually begins between the third to fifth days of disease and characterized by life-threatening hemorrhagic syndrome. The mortality rate ranges from 3 to 80% in different geographic areas [1–4]. This may be associated with the route of transmission, amount of the inoculum, viral strain, and early diagnosis. The treatment options for CCHF are limited and are based on general supportive measures. Ribavirin inhibits replication of the CCHFV *in vitro*, but its efficacy for human therapy remains controversial [1–3].

Currently, there are no licensed vaccines available for CCHFV. The absence of a susceptible animal model for CCHFV infection was severely hindered work on the development of vaccines. Newborn mice succumb to infection but they cannot be used to assess vaccine efficacy due to not similarity to human CCHF and their immature immune systems [9]. Recently, several animal models of CCHFV infection have been developed and used to assess vaccine efficacy [9–14]. Different approaches have been attempted towards the development of a CCHFV vaccine. These include modified vaccinia Ankara–based vaccine, recombinant subunit vaccines, cell culture-based inactivated vaccine, virus replicon particle (VRP) vaccine, gene-based vaccine platforms (DNA or mRNA), virus like particles (VLP), vesicular stomatitis virus-based vaccine and human adenovirus 5-vectored vaccine [15–26]. These vaccines have been demonstrated variable efficacy in mice models for CCHFV. To date, the only CCHFV vaccine tested in humans is the suckling mouse brain-derived vaccine used only in Bulgaria [27]. The suckling mouse brain vaccine was developed in 1970 in the Soviet Union. This isolated virus was inactivated by the use of chloroform, heated at 58°C, separated and absorbed on Al (OH) gel [27,28]. It was approved and licensed in Bulgaria and have been used in military and medical personnel and people living in endemic regions [27,28]. However, this vaccine has not been approved for use in other countries due to possible autoimmune and allergic responses. Currently, the Bulgarian mouse brain derived vaccine is produced by BulBio- NCIPD Ltd, and is prepared using CCHFV strain V42/81, isolated from a patient in 1981 [29]. The Bulgarian Ministry of Health reported that a decrease in the number of cases was observed, from 1105 cases during 1953–1974 to 279 during 1975–1996 [28,29]. However, the vaccination may not be only reason a four-fold reduction in the number of CCHF cases since other factors such as changing ecology and epidemiology of CCHF may also be involved in gradually decreasing of the cases. Data on the immunogenicity of the Bulgarian mouse brain derived vaccine were limited. However, in 2012 a study showed that repeated vaccinations with the Bulgarian mouse brain derived vaccine in healthy volunteers elicited high levels of CCHFV antibodies and anti-CCHFV-spesific T-cell activity but they had low level neutralization activity [30]. There are no experimental data on the immunogenicity and protective efficacy of the Bulgarian mouse brain derived vaccine in mice due to lack of suitable animal models in the past.

We previously showed that a cell culture based vaccine against CCHFV induced neutralization antibody and elicited a significant level of protection against a high dose challenge with homologous CCHFV Turkey-Kelkit06 strain in interferon α/β-receptor knockout (IFNAR^-/-^) mice. In this study, we have used the transiently immune-suppressed (IS) mouse model to explore the immunogenicity and vaccine efficacy of the cell culture based and the mouse brain derived inactivated vaccines against CCHFV. This is the first comparison of the cell culture based and the mouse brain derived inactivated vaccines against CCHFV in the IS mouse model.

## Methods

### Ethics statement

This study was approved by the Committee for Ethics on Animal Experiments (EUHADYEK/EU approval number 14/160) and the Committee for Animal Biosafety Level 3 Research (ERAGEM/EU protocol IP-3–14) in Erciyes University. All of the studies were conducted under the guidelines for animal experiments, were performed as specified in the regulation 5199 which describes animal protection and working with laboratory of animals in Turkey.

### Animals

Six to nine weeks old female Balb/c mice used in all experiments. Balb/c mice were obtained from Kobay, Turkey. Experimental animals were fed with standard rodent pellets supplemented with grain and given water ad libitum. Throughout this study, the animals were housed in an Isocage system (Allentown Inc. USA) with climate-controlled (22±2°C; relative humidity, 65%) and photoperiod controlled (12-hours light-dark cycles). Only healthy mice were included in the study. All animal experiments were performed in an Animal Biosafety Level 3 (ABSL-3) enhanced facility in Vaccine Research Development and Application Center (ERAGEM) in Erciyes University.

### Cell, virus and antibodies

Vero E6 cells (African green monkey kidney) obtained from ATCC (CRL 1586) were maintained in Dulbecco’s modified Eagle’s medium (DMEM) supplemented with 10% heat-inactivated fetal bovine serum (FBS), 100 mM L-glutamine, 50 U/ml penicillin, 50 μg/ml streptomycin (Sigma–Aldrich, Germany). CCHFV Turkey-Kelkit06 strain was isolated from the blood of a patient in Turkey [31]. The CCHFV Turkey-Kelkit06 strain was passaged three times by intracerebral inoculations of 2–3 days old suckling mice. The viral stocks were prepared on Vero E6 cells by infection of T150 cell culture flasks with a 1:100 dilution. Supernatants were collected on days 5 post-infection, cleared of cell debris by centrifugation and aliquots were stored at -80°C. The titer of the viral stock was determined by a pseudo-plaque assay (PPA) [32]. All handling of virus was conducted in a biosafety level 3 enhanced facility (BSL-3). Polyclonal rabbit and mouse anti-CCHF virus sera were generated in rabbits and mice as described previously [31].

### Vaccine preparation

The cell culture based inactivated vaccine against CCHFV was prepared as described previously with minor modifications [18]. Confluent Vero E6 cell monolayers in T150 flasks were infected with a 0.01 multiplicity of infection (MOI) of CCHFV Turkey-Kelkit06 strain and incubated for 1 hour at 37°C with rocking at 10 min intervals. The inoculum was discarded, and cells were washed twice with PBS and fed with DMEM containing 2% FBS. Vero E-6 cells infected with the CCHFV Turkey-Kelkit06 strain showed 70–80% CPE at 5 day post-inoculation. At this point, infected cells were harvested and lysed during freeze-thaw cycles twice, after centrifugation the bulk culture of the virus was precipitated with 0.5M NaCl, and 10% (w/v) PEG8000 and the solution stirred overnight at 4°C.

The mixture was centrifuged at 8000 g for 30 min at 4°C. The supernatant was discarded, and the virus precipitate was dissolved in TNE buffer (50 mM Tris-HCl, 150 mM NaCl, and 5 mM EDTA, pH 7.2). The virus-containing solution was loaded on a 20–60% sucrose gradient and centrifuged in a Beckman SW32 rotor at 30 000 rpm at 4°C. Five hundred microliters of fractions were collected from the bottom of the tube by the fraction collector. The fractions were analyzed for the CCHFV proteins by western blotting. The virus pool was diluted four-fold with PBS and subjected to ultracentrifugation at 24 000 rpm at 4°C. Each virus pellet was diluted in five hundred microliters of PBS. For inactivation of the virus, formalin (37% formaldehyde, Sigma, USA) was diluted 1:40 and was added dropwise to the purified virus pool to give a final formaldehyde dilution of 1/2000. The inactivation procedure was carried out for 168 hour at 22°C. The residual formalin in the samples was neutralized by the addition of one part 3.75% (w/v) sodium metabisulfite to 100 parts of the virus suspension. The neutralized virus solution was dialyzed at 4°C against PBS. After the neutralization step, the inactivated virus suspension was filtered through a 0.22μm low protein binding filter unit (Millipore, USA). The absence of infectious virus was confirmed by intracerebrally inoculation of the vaccine antigen into 2 to 3 days old Balb/c suckling mice. PPA was also performed for reconfirmation of the absence of live virus. The bulk vaccine was mixed with adjuvant (Imject Alum, Pierce, USA) at a concentration of 250 μg of alum per 500 μl of vaccine dose.

CCHFV Turkey-Kelkit06 strain was passaged 3 times in 2 to 3 days old Balb/c suckling mice and used as seed virus for vaccine preparation. The brains were harvested after animals showed typical signs of paralysis. PBS containing 5 mM ethylene diamine tetraacetate (EDTA) was added to the brains (1.5 ml/brain), which were then homogenized and kept at -80°C for overnight. The mixture was freeze-thawed twice, after centrifugation the mixture containing the virus was precipitated with 0.5 M NaCl, and 10% (w/v) PEG8000 and the solution stirred overnight at 4°C. The mixture was centrifuged at 8000 g for 30 min at 4°C. After concentration of the brain tissue, the solution was subjected to protamine sulphate precipitation (Merck Germany). The virus-containing mixture was loaded on a 20–60% sucrose gradient and centrifuged in a Beckman SW32 rotor at 30 000 rpm at 4°C. Five hundred microliters of fractions were collected and analyzed for the CCHFV proteins by western blotting. The virus pool was diluted four-fold with PBS and subjected to ultracentrifugation at 24 000 rpm at 4°C and the virus pellet was dissolved in five hundred microliters of PBS. To prepare the inactivated mouse brain derived vaccine, formalin was diluted 1:40 and was added to the purified virus pool to give a final formaldehyde dilution of 1/2000. The inactivation procedure was performed for 168 hour at 22°C. After neutralization of the residual formalin, the virus solution was dialyzed at 4°C against PBS and was filtered through a 0.22μm low protein binding filter unit (Millipore, USA). The virus inactivation was confirmed by intracerebrally inoculation of the vaccine antigen into 2 to 3 days old Balb/c suckling mice. PPA was also done for reconfirmation of the absence of live virus. The bulk vaccine was mixed with adjuvant (Imject Alum, Pierce, USA) at a concentration of 250 μg of alum per 500 μl of vaccine dose.

### SDS-PAGE and immunoblotting

The cell culture based and the mouse brain derived vaccines antigens were separated on 10% resolving and 5% stacking SDS-PAGE gels in a mini electrophoresis unit (Bio-Rad, USA) at 90 V for 2 hour. The protein gel was directly stained with 0.05% Coomassie brilliant blue (Sigma, USA) or the proteins were transferred onto a nitrocellulose membrane (Millipore, USA) in wet conditions using a trans-blot apparatus (Bio-Rad, USA). After blocking with 5% skimmed milk the membrane was incubated with a primary antibody followed by a goat anti-rabbits alkaline phosphatase (AP)-conjugated antibody (1:2000 dilution, Southern Biotech, USA). The membrane was reacted with the ECL substrate solution (Pierce ECL, USA) and exposed to an autoradiograph film (Sigma, Germany) and developed using a Kodak developer (Sigma, Germany).

### Immunogenicity of the cell culture based and the mouse brain derived vaccines

Groups of 10 Balb/c mice were vaccinated intraperitoneally with 5, 10, or 20μg of either the cell culture based vaccine or the mouse brain derived vaccine. The control group (n = 7) was mock immunized with PBS. Booster injections with the same formulation were given on days 14 and 27 after the first immunization. Blood were collected via submandibular bleeds before each vaccination.

### Titration of neutralization antibody

The neutralization titer (NT) of the sera collected from the animals was determined in triplicate using a Focus Reduction Neutralization assay (FRNT) as described earlier with minor modifications [33]. Briefly, Vero E6 cells were grown to confluence with DMEM containing 10% FBS in 24-well microtiter plates (Corning, USA) at 37°C, 5% CO_2_ for 18–24 hour. The sera from the immunized and control mice were heat-inactivated and serial 2-fold dilutions were prepared and mixed with an equal volume of the CCHFV Turkey-Kelkit06 strain-containing 50 pseudo plaque-forming units. The plates were incubated for overnight at 4°C. Two hundred microliters of the serum–virus mixtures were adsorbed to confluent cell monolayers (in triplicate) and incubated for an additional hour at 37°C. The supernatant was removed, and the cell monolayer was overlaid with the virus medium (DMEM with L-glutamine containing 2% FBS, 2% HEPES (1 M), and 1% penicillin, streptomycin 100×) supplemented with 1% carboxymethyl cellulose (Sigma-Aldrich, Germany) and incubated at 37°C, 5% CO_2_ for 4 days. The cells were fixed with formaldehyde (Sigma-Aldrich, Germany) and permeated with 0.1% Triton X 100 in PBS for 20 min and blocked with 5% skim milk in PBS. The polyclonal rabbit anti-CCHFV serum (1:1500) was added to each well in TBST (100 mM Tris-HCl pH8.0, 1.5 M NaCl, 1% Tween 20) and incubated for 1 hour at room temperature. After three washes for 10 min in TBST, goat anti-rabbit conjugated with fluorescein isothiocyanate (FITC) was diluted (1/1000) with TBST and overlaid the cells for 1 hour at room temperature. The cells were washed five times with TBST, the cells were overlaid by PBS and glycerin (Sigma) for checking through fluorescence microscope. The neutralizing anti-CCHFV antibody titers were directly assigned to the highest dilution with > 50% reduction. The results were recorded as the geometric mean titer (GMT) ± the standart error (S.E.).

### ELISA

The viral antigens prepared from Vero E6 cells infected with the Turkey-Kelkit06 strain of CCHF virus were purified by sucrose gradient ultracentrifugation. After inactivation with formaldehyde, the antigen samples were dialyzed at 4°C against PBS. The antigen suspension was filtered through a 0.45μm low protein binding filter unit (Millipore, USA). Flat-bottomed 96-well plates (Nunc, Denmark) were coated with 1 μg/well of the viral antigen in 0.05 M carbonate bicarbonate buffer (pH 9.6) at 4°C for overnight. The plate was blocked with 5% skim milk in PBS for 1 hour at 37°C. The plate was incubated at 37°C for 1 hour with sera from the immunized mice serially diluted in PBS containing 1% skim milk and incubated at 37°C for 1 hour with a horseradish peroxidase (HRP)-conjugated polyclonal goat anti-mouse IgG (Southern Biotech, USA) diluted 1:5000. The plate was colorized with the peroxidase substrate solution and the reaction was stopped by 1N HCl. The absorbance was read at a wavelength of 450 nm (OD_450_) by a spectrophotometer (Biotek ELx80, Germany). The end point of the antibody titer was determined by an absorbance greater than the mean + 2 standard deviation of the negative control sera. The results were recorded as the geometric mean titre (GMT) ± the standard error (S.E.).

### IFN-γ ELISPOT assay

The cell culture based and the mouse brain derived vaccines spesific T cell responses were analyzed by quantification of IFN-γ production from splenocytes by ELISPOT. Groups of 6 Balb/c mice were vaccinated intraperitoneally with 20μg of either the cell culture based vaccine or the mouse brain derived vaccines on day 1, day 14 and day 28. Two weeks after third immunization (day 42) the plate was blocked with RPMI 1640 medium (Gibco) for 1 hour before the addition of the splenocytes. 2,5×10^5^ freshly isolated mouse splenocytes were added to each well, and then stimulated with MOI 0.1 of purified live CCHFV Turkey-Kelkit06 strain. Vero E6 mock cell culture was used as a negative control. Splenocytes incubated in culture media alone served as a background control and splenocytes stimulated with 10 μg/ml of concanavalin A (Sigma) as a cell viability control.

The splenocytes were stimulated and cultured at 37°C for 18 hours. After the removal of cells, the plate was incubated with a biotinylated anti-mouse IFN-γ detection antibody (Mabtech) diluted in 0.5% FBS PBS (0.2μg/well) for 2 hours, followed by adding 1/1000 diluted AP-conjugated streptavidin (Mabtech) for 1 hour. The plate was colorized by adding TMB substrate (Mabtech) to image the IFN-γ-secreting cells. Stereo microscope was used to count the spots. Results were presented by spot forming unit (SPU) per milliliter. Results are the mean of three experiments.

### Assessing the lethality Balb/c mice treated with MAb-5A3 by CCHFV Turkey-Kelkit06 strain

Balb/c (n = 4/group) mice were inoculated intraperitoneally with MAb-5A3 (Leinco Technologies Inc) 24 hour prior to (2.0 mg) and 24 hour after (0.5 mg) CCHFV Turkey-Kelkit06 strain challenge. Balb/c mice were injected intraperiteonally with containing 1000, 100 and 10 Pseudo Plaque Forming Unit (PPFU) of the virus. The mice were monitored twice daily for clinical score, body temperature, weight change and survival. Animals were scored for the each parameter as follows: normal (0), ruffling (1), ruffling and slight lethargy (2), lethargy (3), labored breathing and immobility (4). Mice showing clinical score 4 were considered to have reached the experimental end point and were euthanized.

### Protective efficacy studies in transiently immune-suppressed Balb/c mice

Groups of 10 Balb/c mice were vaccinated intraperitoneally with 20μg of either the cell culture based or the mouse brain derived vaccines. The control group (n = 7) was mock immunized with PBS. Booster injections with the same formulation were given on days 14 and 27 after the first immunization. Mice were challenged with 100 PPFU of CCHFV Turkey-Kelkit06 strain by intraperitoneal injection. MAR1-5A3 (mouse anti- mouse IFNAR, IgG1) were administered at 2 mg per mouse 24 hour before challenge and 0.5 mg at 24 hour after challenge. The mice were monitored daily for clinical score, body temperature, weight change, and survival.

### Statistics

All graphics and statistical data analysis were performed by using GraphPad Prism 7 software (GraphPad, USA). Weight loss significance was determined using ordinary one-way ANOVA. Kaplan-meier curve was used to show survival graph and log rank test (Mentel-Kox) was performed for survival statistics. Two-way ANOVA was utilized to determine the statistical differences between the groups in ELISA, neutralizing antibody assay and viral titers of vaccinated animals. Mann-Whitney U test was used to compare between vaccinated groups in ELISPOT assay. For all the statistical analysis, significance levels were set *p* value less than 0.05 where *p<0.05, **p<0.005, ***p<0.0005, ****p<0.0001.

## Results

### Antigenicity of the cell culture based and the mouse brain derived vaccines against CCHFV

**Fig 1:**
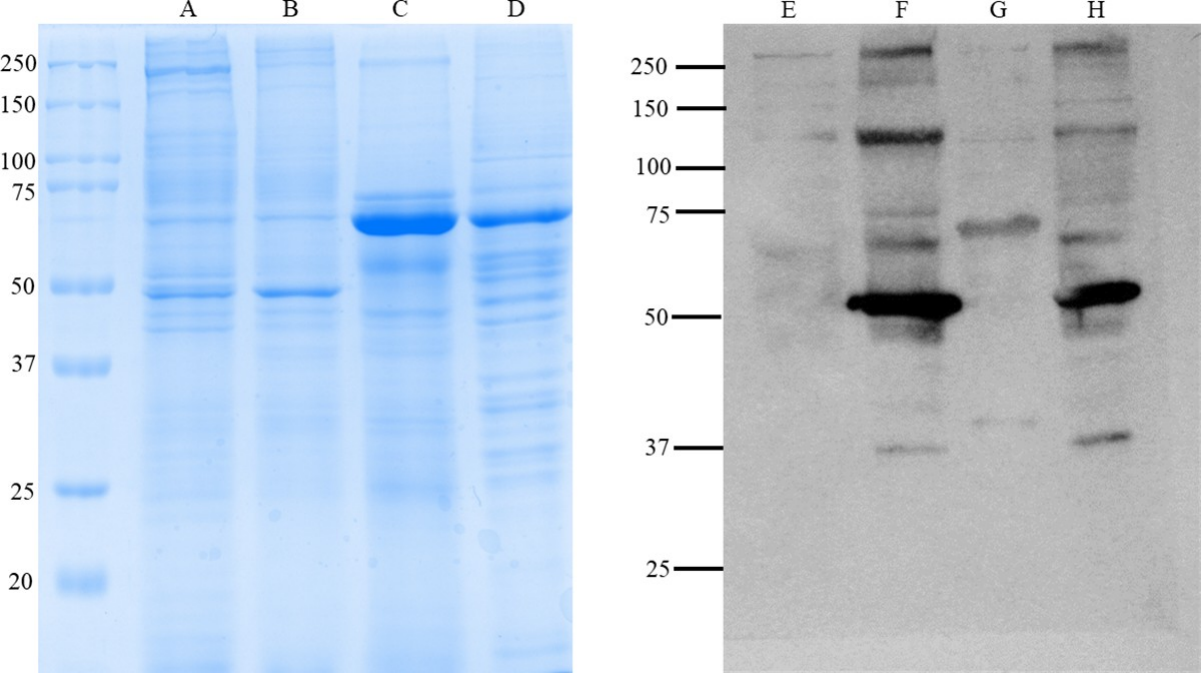
Confirmation of the antigenicity of the cell culture based and the mouse brain derived vaccines by SDS-PAGE and western blotting.

The antigenicity of the cell culture based and the mouse brain derived vaccines was determined by SDS-PAGE and blotting. The purified cell culture based (Fig 1A), mouse brain derived vaccine antigens (Fig 1B), the purified mock infected Vero E6 cells (lane C), and the purified mock infected mouse brain (lane D). were electrophoresed on SDS-PAGE gel and immunoblotted with CCHFV Turkey-Kelkit06 strain polyclonal antibody to visualize the antigenic bands. The viral proteins NP (55 kDa), Gn (37 kDa), and Gc (75 kDa) were present in the cell culture based (Fig 1F) and the mouse brain derived (Fig 1H) vaccine antigens, indicating that two vaccine preparations represented almost identical antigenicity. A high MW band >250kda have been detected not only two vaccine preparations but also mock infected Vero cells (Fig 1E) and mock infected mouse brain (Fig 1G). We also observed a band between 100 and 150kda in the mock infected samples and the two vaccine preparations (Fig 1E–1H). Some protein aggregates, residual host cell proteins and serum used in the culturing media may all contribute to the background noise.

SDS-PAGE separation of proteins in the cell culture based and the mouse brain derived vaccine preparations. Each preparation contains 20μgr of the antigens. The purified cell culture based vaccine antigens (lane A), the purified mouse brain derived vaccine antigens (lane B), the purified mock infected Vero E6 cells (lane C), and the purified mock infected mouse brain (lane D). The purified cell culture based vaccine antigens (lane F), the mouse brain derived vaccine antigens (lane H) and mock infected Vero E6 cells (lane E) and mock-infected mouse brain (lane G) were immunoblotted with 1:2000 dilution of the hyperimmune serum grown in rabbit against the CCHFV Turkey-Kelkit06 strain and followed by goat anti-rabbit AP conjugated antibody to visualize the antigenic bands.

### Comparison of antibody responses to the cell culture based and the mouse brain derived vaccines in Balb/c mice

**Fig 2:**
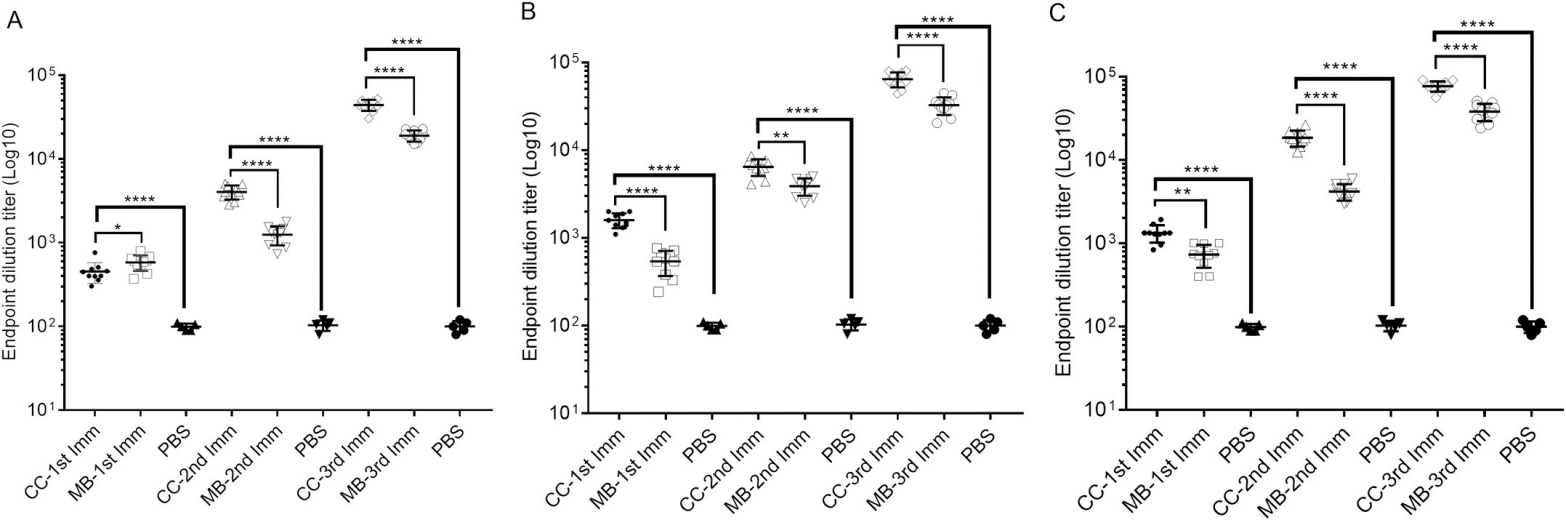
CCHFV-spesific IgG response to the cell culture based and the mouse brain derived vaccines against CCHFV in Balb/c mice. Indirect ELISA measuring CCHFV-spesific IgG responses in mice (ten mice per group analyzed) sera immunized with 5μg (A) 10μg (B) and 20μg (C) the cell culture based and the mouse brain derived vaccines with alum. Booster doses were given on days 14 and 28 after the first immunization. PBS is a mock control group of mice mock-immunized with buffer only. The sera obtain from the mice at different time points (14, 28 and 42 days) were assayed for anti-CCHFV antibody end point titre. Two way ANOVA test was performed to determine the differences between groups in ELISA titers where p<0.05 considered significant. Error bars represent mean ± the standard deviation (SD). *p<0.05, **p<0.005, ***p<0.0005, ****p<0.0001. CC- cell culture based inactivated vaccine, MB- mouse brain derived inactivated vaccine, 1st Imm- first immunization, 2nd Imm- second immunization, 3rd Imm- third immunization and PBS (Phosphate-buffered saline).

**Fig 3:**
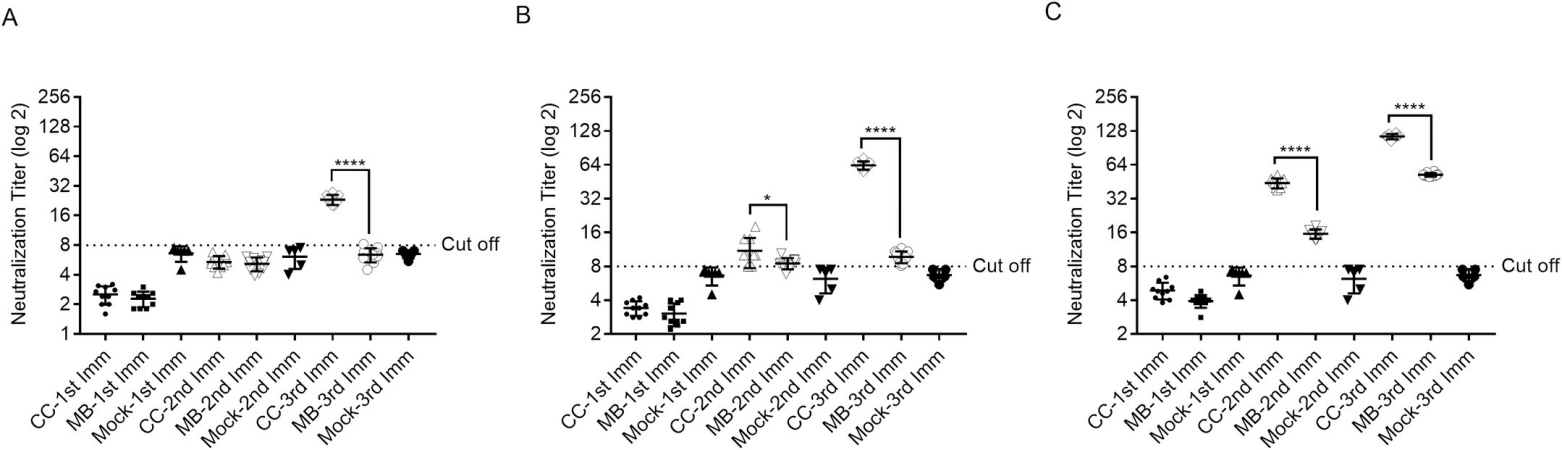
CCHFV-spesific neutralizing antibody responses to the cell culture based and the mouse brain derived vaccines against CCHFV in Balb/c mice. Neutralizing antibody responses of mice vaccinated with 5μg (A), 10μg (B) and 20μg (C) of the cell culture based and the mouse brain derived vaccines with alum. All of the groups received primary immunizations and two booster immunizations 2 weeks apart. PBS is a mock control group of mice mock-immunized with buffer only. The sera obtain from vaccinated mice at different time points (14, 28, 42 days) were assayed for neutralizing antibody titers that were determined by a 50% Focus Reduction Neutralization Test (FRNT). Two way ANOVA test was used to determine the significance of the data between the groups in neutralizing antibody titers where p<0.05 considered significant. Error bars represent mean ± the standard deviation (SD). The dotted line illustrates the limit of detection of neutralization titer. *p< 0.05, ****p< 0.0001. CC- cell culture based inactivated vaccine and MB- mouse brain derived inactivated vaccine, 1st Imm- first immunization, 2nd Imm- second immunization, 3rd Imm- third immunization and PBS (Phosphate-buffered saline).

To determine the antibody responses of the cell culture based and the mouse brain derived vaccines against CCHFV Turkey-Kelkit06 strain, Balb/c mice were immunized intraperitoneally with the two vaccine preparations containing 5μg (Fig 2A), 10μg (Fig 2B), 20μg (Fig 2C) and booster doses were given at 2 and 4 weeks after the first immunization. The first immunization of mice with the cell culture based and the mouse brain derived vaccines induced low, but detectable, levels of antibodies as measured by indirect ELISA (Table 1). The levels of virus-spesific antibodies were significantly increased following the booster immunizations in all groups. The serum antibody titer induced by the cell culture based vaccine at all three concentrations was significantly higher than the titer obtained from the mice immunized with the mouse brain derived vaccine at 4 weeks after the first immunization (Fig 2A–2C and Table 1). The first immunization of mice with the cell culture based and the mouse brain derived vaccines containing 5μg (Fig 3A), 10μg (Fig 3B) and 20μg (Fig 3C) dose groups did not generate any detectable neutralization antibodies (Table 1). The booster dose given at 2 weeks after the first immunization induced neutralization antibodies in mice immunized with the cell culture based and the mouse brain derived vaccines containing 20μg doses (Fig 3C and Table 1) but the 5μg and 10μg dose groups of the two vaccine preparations were either zero or borderline positive, respectively (Fig 3A and 3B and Table 1). The third immunization was given on 28 days after the first immunization, the levels of neutralization antibody titers were significantly increased in all groups (Fig 3A–3C) whereas 5μg and 10μg dose groups from the mouse brain derived vaccine was either zero or borderline positive, respectively (Fig 3A and 3B and Table 1). Our findings indicated that CCHFV-spesific antibody titres induced by two vaccine preparations increased in a dose-dependent manner. The CCHFV-spesific IgG were detected following the first dose but repeated doses were required to induce neutralization antibodies against CCHFV. Cumulatively these data suggest that the cell culture based vaccine exhibited higher antigenicity than the mouse brain derived vaccine.

**Table 1:** Comparison of the serological responses in Balb/c mice intraperitoneally immunized with the cell culture based and the mouse brain derived vaccines against CCHFV. Booster doses were given at 2 and 4 weeks after the first immunization, and virus-spesific antibodies in mice were assessed at 14, 28, and 42 days post-vaccination.

Vaccine	Dose (μg)	Antibody titre (GMT^a^)					
		First Immunization		Second Immunization		Third Immunization	
		ELISA	*N ab	ELISA	N ab	ELISA	N ab
**Cell culture based**	20	1333.3± S.D	**ND	18466.7± S.D	44± S.D	76920± S.D	114.3± S.D
	10	1596.2± S.D	ND	6468± S.D	11± S.D	64533± S.D	63.1± S.D
	5	451.4± S.D	ND	4041± S.D	ND	44200± S.D	23.3± S.D
**Mouse brain derived**	20	733.3± S.D	ND	4200± S.D	15.5± S.D	38133± S.D	52.1± S.D
	10	540.8± S.D	ND	3900± S.D	8.5± S.D	32653± S.D	9.7± S.D
	5	581.2± S.D	ND	1251± S.D	ND	19000± S.D	ND

^a^The end point antibody titre represented as the mean ± S.D. of ten animals.

* N ab; Neutralization antibody titre

**ND: Not determined

The presence of a CCHFV-spesific antibodies in vaccinated mice from both vaccine preparations (20μg) were also tested by immunoblot analysis after two weeks of the last immunization. Immunoblotting analysis of the serum pools from the vaccinated animals reacted with the viral antigens of NP (53 kDa), Gc (75 kDa) and Gn (37 kDa) (S1 Fig).

### Evaluation of IFN-γ response to the cell culture based and the mouse brain derived vaccines in Balb/c mice

**Fig 4:**
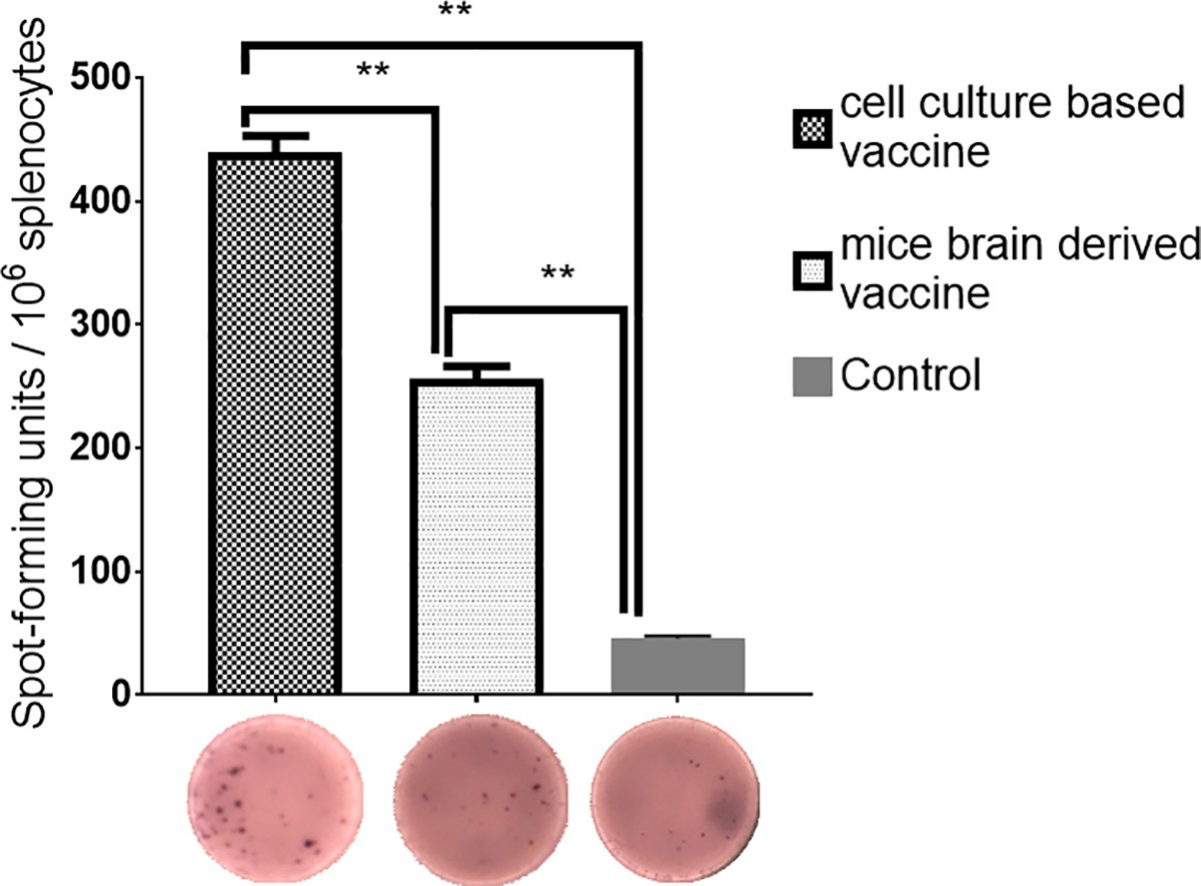
CCHFV-spesific IFN-γ response to the cell culture based and the mouse brain derived vaccines against CCHFV in Balb/c mice. Balb/c mice were immunized three times at two weeks intervals with 20μg of the two vaccine preparations or PBS control. Splenocytes isolated from Balb/c mice (*n* = 6, pooled) were stimulated with MOI 0.1 of purified live CCHFV for 18 hour. The number of IFN-γ producing cells was measured by ELISPOT assay. Results are the mean of three experiments. Mann-Whitney U test used to compare between two vaccinated groups. **p< 0.005.

To characterize the immunological properties of the cell culture based and the mouse brain derived vaccines, Balb/c mice were immunized with the two vaccine preparations containing 20μg and booster doses were given on 14 and 28 days after the first immunization. On day 42, splenocytes from the vaccinated groups were collected aseptically and IFN-γ ELISPOT assay performed to measure the cellular immune response. For both the vaccine groups, T cell responses were detected, and the cell culture based vaccine induced significantly higher (p< 0.005) IFNγ responses than that of mouse brain derived vaccine: between 140 and 176 spots/2,5x10^5^ cells in the cell culture based vaccine vs. 98–119 spots/2,5x10^5^ cells found in the mouse brain derived vaccine (Fig 4). These results indicated that both the cell culture based and the mouse brain derived vaccines elicited T-cell-mediated immune response.

### Vaccination and challenge studies

**Fig 5:**
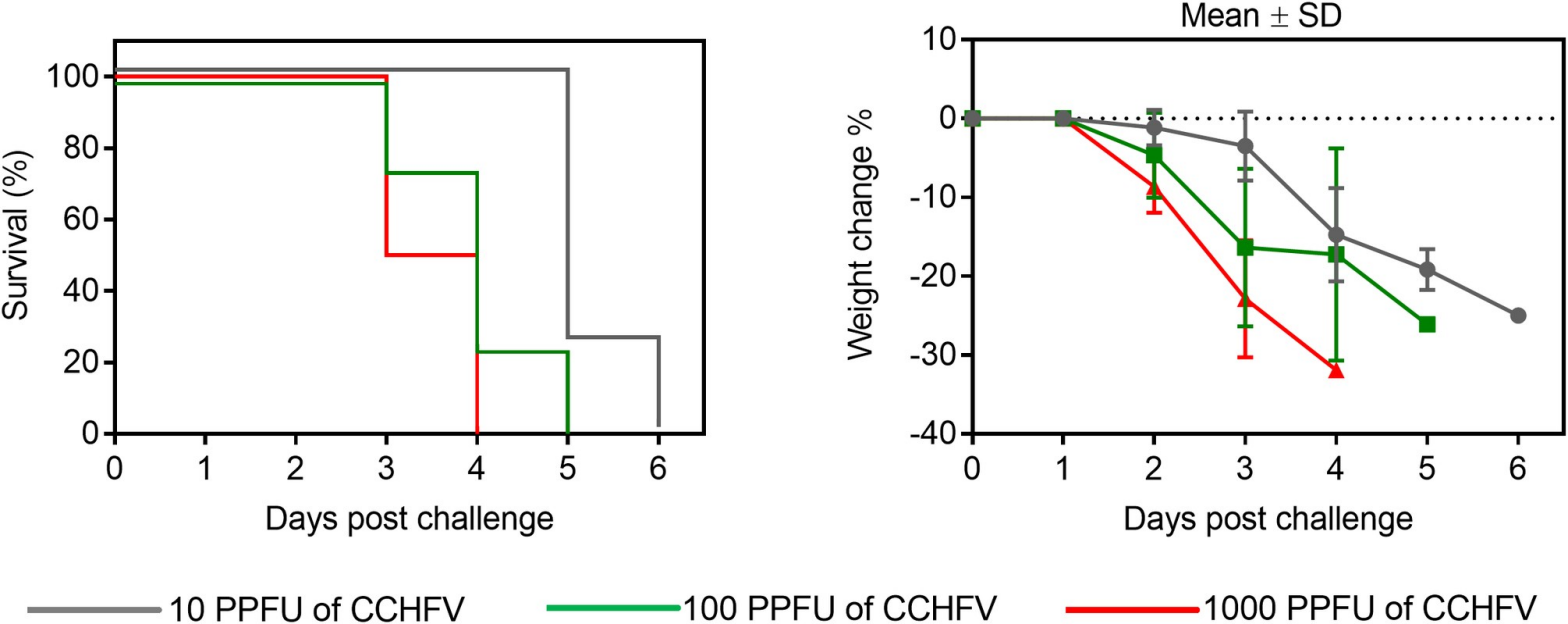
Evaluating the lethality Balb/c mice treated with MAb-5A3 by CCHFV Turkey-Kelkit06 strain. Four Balb/c mice were injected intraperitoneally with MAb-5A3 24 hour prior to (2.0 mg) and 24 hour after (0.5 mg) CCHFV Turkey-Kelkit06 strain challenge. The IS Balb/c were received with containing 1000, 100 and 10 Pseudo Plaque Forming Unit (PPFU) of the virus. The mice were monitored twice daily for weight change and survival. The standard deviations are shown as error bars.

To asses the lethality of Balb/c mice treated with MAb-5A3 by CCHFV Turkey-Kelkit06 strain, we adjusted a recently developed model that transiently blocks type I interferon activity by an antibody to the IFN-α/β receptor (MAb-5A3) [13]. Briefly, Balb/c mice were received to MAb-5A3 one day before to (2.0 mg) and one day after (0.5 mg) CCHFV challenge with 10 PPFU, 100 PPFU, and 1000 PPFU by intraperitoneal route. All mice exhibited weight lose (Fig 5B). The general conditions deteriorated rapidly and all challenge doses lead to 100% lethality between 3 and 6 days (Fig 5A). We found that the IS Balb/c mice were very susceptible to the CCHFV Turkey-Kelkit06 strain, suggesting that the IS mouse CCHFV model could be used for CCHFV vaccine efficacy studies.

**Fig 6:**
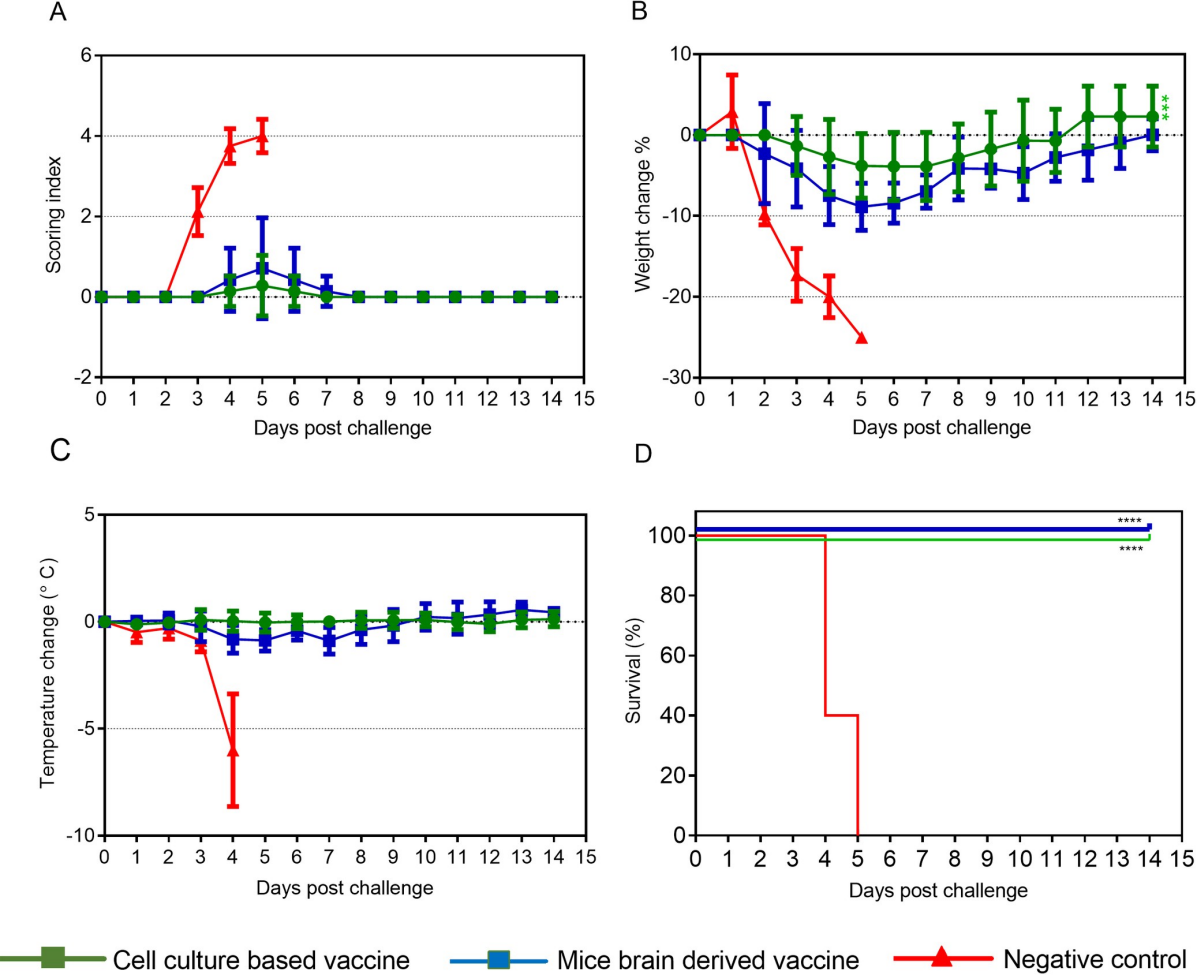
Protection of the IS Balb/c mice immunized with the cell culture based and the mouse brain derived vaccines. Groups of 10 IS Balb/c mice were immunized three times at two weeks intervals with 20μg of the two vaccine preparations. The control group of the IS Balb/c mice (n = 7) was mock immunized with PBS. All animals were challenged with 100 PPFU CCHFV Turkey-Kelkit06 strain two weeks after the last immunization. The mice were monitored twice daily for the cumulative mean symptom scores (A), the daily variations in weight as percentages compared to before the virus challenge (B), body temperature (C), and geometric mean time to death and survival (D). The animals were monitored for three weeks after the challenge. The standard deviations are shown as error bars. Log rank (Mantel-Kox) test was utilized for survival statistics, **** p<0.0001. A one-way ordinary ANOVA was used to analyse weight loss over time between two vaccinated groups, ***p<0.0005.

We next examined to the protective efficacy of the cell culture based and the mouse brain derived vaccines in immunocompetent Balb/c mice. Based on the neutralization titers obtained from different dose groups immunized with the cell culture based and the mouse brain derived vaccines (Table 1), Balb/c mice were vaccinated with the two vaccine preparations containing 20μg with three doses, 100 PPFU of CCHFV Turkey-Kelkit06 strain was subsequently used for challenge studies in the IS mouse CCHFV model. After two weeks of the last immunization, the vaccinated mice were injected intraperiteonally with the MAb-5A3 one day before and one day after challenge with 100 PPFU by intraperitoneal route as described in Methods. The IS Balb/c mice in control group showed signs of the disease, displayed as rapid weight loss at 2 days post-challenge (Fig 6A and 6B, respectively). They became hypothermic at 4 days post-challenge and died or reached the experimental end point between days 4 and 5 post-challenge and were euthanized (Fig 6C and 6D, respectively). The cell culture based and the mouse brain derived vaccines provided full protection against challenge with 100 PPFU of the virus in the IS Balb/c mice (Fig 6D). All vaccinated animals exhibited no clinical signs and no significant changes in body temperature (Fig 6A–6C, respectively). However, animals immunized with the mouse brain derived vaccine lost 8,8% of their original body weight between days 4–6 post-challenge, whereas weight loss of the mice immunized with the cell culture based vaccine was around 4,5% between days 4–6 post-challenge (Fig 6B). All animals immunized with the cell culture based and the mouse brain derived vaccines began gaining weight at 7 days post challenge and returned to their starting weight by day 11 and 14 after challenge, respectively (Fig 6B).

**Fig 7:**
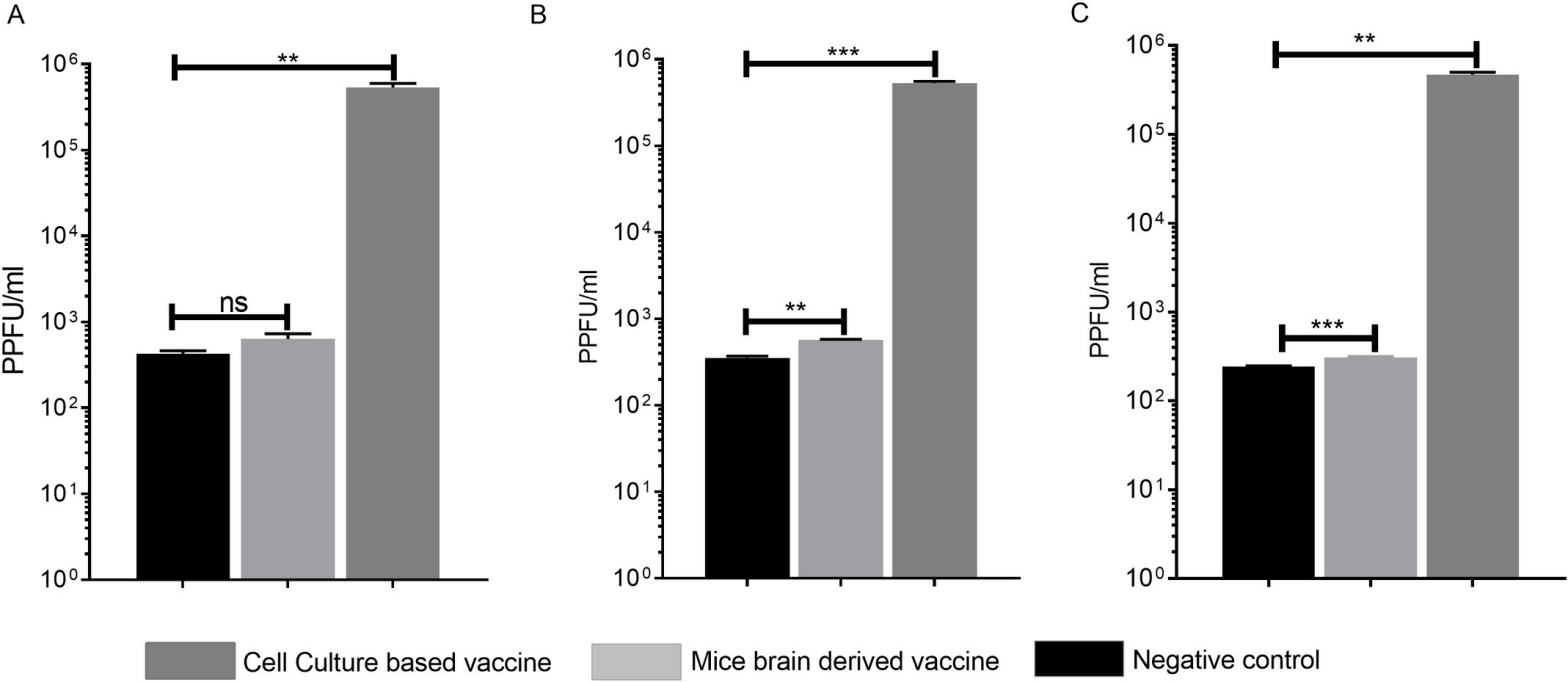
Determination of virus titers of the vaccinated animals and challenged mice. Groups of 3 Balb/c mice were immunized three times at two weeks intervals with 20μg of the cell culture based and the mouse brain derived vaccines. The control group of Balb/c mice (n = 3) was mock immunized with PBS. The IS Balb/c mice were challenged with 100 PPFU of CCHFV Turkey-Kelkit06 strain two weeks after the last immunization. All animals were sacrificed at 3 days post-infection. The virus was isolated from the blood (A), liver (B) and spleen (C) as described in the Methods. The standard deviations are shown as error bars. Two way ANOVA test was used to determine significant differeces between the groups in virus titers of vaccinated animals. **p<0.005, ***p<0.0005, ns: non-significant.

At 3 days post-challenge, animals from the two vaccine groups and control group (n = 3 each) were euthanized and their liver, spleen and blood were sampled for virus titrations. The mean virus titers in blood from the animals immunized with of the cell culture based vaccine were similiar to those detected in the mouse brain derived vaccine (Fig 7A), whereas liver and spleen viral titers on day 3 post-infection of animals vaccinated with the mouse brain derived vaccine were higher to those detected in the cell culture based vaccine (Fig 7B and 7C). The infectious titers of the vaccinated animals were approximately 3 log lower than the unvaccinated animals, indicating that both the cell culture based and the mouse brain derived vaccines signicantly reduced the viral titers (Fig 7A–7C).

## Discussion

CCHF is one of the vector borne diseases that causes severe hemorrhagic disease in humans. Due to its high mortality rate, lack of the effective treatment options, and expanding new foci of the CCHF endemic area, CCHFV has been included on the World Health Organization Research and Development Blueprint list of infectious agents [34]. Therefore, development of safe and effective vaccines against CCHFV is a high priority for preventing disease in endemic countries.

Historically, the only lethal animal model was infection of newborn mice but they do not accurately recapitulate human CCHF disease [9]. However, two models established CCHFV lethality in immunocompromised adult mice. A129 and C57BL/6 mice lacking the type IFN receptor A (IFNAR^-/-^) could support a severe and lethal disease model [11]. The second model was STAT-1^-/-^ mice which have deficiencies in both IFN-I and type II interferon (IFN- γ) signaling [10]. The knockout mouse models which recapitulate some of the clinical features of CCHF in humans has allowed for the evaluation of newly developed vaccine candidates against CCHFV [35]. We previously developed a cell culture based inactivated CCHFV vaccine that demonstrated protective efficacy in IFNAR^-/-^ mice against a lethal infection with the homologous strain of CCHFV [18]. Vaccination with the attenuated poxvirus vector modified vaccinia virus Ankara expressing CCHFV glycoproteins (MVA-GP) can protect IFNAR^-/-^ mice against CCHFV challenge [15]. With the similar approach, the whole genome of M segment of CCHFV was cloned into a human serotype 5 adenovirus vector. Vaccination of STAT-1^-/-^ mice with the adenovirus-vector expressing CCHFV glycoproteins produced anti-CCHFV glycoprotein antibody responses but mice were not protected against challenge [17,36]. Kortekaas et al. also showed that STAT-1^-/-^ mice vaccinated with ectodomains of the structural glycoproteins Gn and Gc produced using a *Drosophila* insect cell–based expression system did not show any protection despite inducing a significant antibody response (17). However, Rodriguez et al. reported that immunization of STAT-1^-/-^ mice with a recombinant vesicular stomatitis virus (rVSV) expressing the CCHFV glycoprotein precursor (GPC) provided 100% protection. They found that anti-CCHFV-GP IgG and neutralizing antibody titers were observed in surviving animals but T cell responses were not evaluated (25). The CCHFV M segment encodes a polyprotein glycoprotein precursor (GPC). After very complex processing, glycoproteins yields envelope glycoproteins Gn and Gc, nonstructural NSM GP160, GP85, and GP38 and mucin-like domain. Even though it is difficult to define the differences in protection between the vaccine approaches, rVSV might drive correct processing of the glycoproteins of CCHFV. Choosing the right animal model for the CCHFV vaccines under investigation is critical in determining protective efficacy. More recently, a novel IS mouse CCHFV model has been developed. MAb-5A3 antibody was used to block IFN-I signaling in immune intact, wild-type mice at the time of CCHFV infection. Garrison and colleagues have concluded that immune competent mice can be used to evaluate CCHFV vaccines and protective efficacy can be examined by transient inhibition of IFN-I using MAb-5A3 proximal to the time of challenge [13]. The advantage of this approach is that it allows immune responses to be elicited in immunologically competent mice with IFN I blockade only induced at the time of infection.

In the present study, we have determined that the IS mice were susceptible to the CCHFV Turkey-Kelkit06 strain, serial dilutions of the virus were inoculated into Balb/c mice treated with MAb-5A3. All challenge doses lead to 100% lethality between 3 and 6 days (Fig 5A). This is in agreement with results of an earlier our study demonstrating that IFNAR^-/-^ mice were susceptible to the CCHFV Turkey-Kelkit06 strain [18]. Then, we evaluated the immunogenicity of the cell culture based and mouse brain derived vaccines in Balb/c mice with three different dose groups. CCHFV-spesific IgG antibodies were observed in all dose groups after the first immunization and significantly increased between the second and third boosting in two vaccine preparations (Fig 2A–2C and Table 1). Although the CCHFV-spesific neutralization titers were significantly higher in the cell culture based vaccinated mice compared with the mouse brain derived vaccinated mice for all dose groups booster doses were required to induce the neutralization acitivity and titers of the neutralization antibodies against CCHFV were dose-dependent manner (Fig 3A–3C and Table 1). Similarly, Mousavi-Jaz et al. showed that individuals vaccinated with the Bulgarian mouse brain derived vaccine showed high levels of CCHFV-spesific antibodies but repeated doses were required to induce neutralization activity against CCHFV [31]. Our data are in agreement with that of Toriniwa et al. in which the immunogenicity of a cell-derived vaccine was higher than that of the conventional mouse brain-derived vaccine against Japanese encephalitis (JE) vaccine [37]. They showed that E protein glycosylation differed between the hosts for viral proliferation (mouse brain cells vs. Vero cells), suggesting that the Vero cell-derived vaccine differs from the mouse brain-derived vaccine regarding viral glycosylation, particularly the glycosylation of E protein, which is considered to be involved in immunogenicity. This might be one of the possible explanation why CCHFV-spesific IgG and neutralization titers were higher in the cell culture based vaccinated mice than those mice receiving the mouse brain derived vaccine against CCHFV.

In this study, both the cell culture based and the mouse brain derived vaccinated the IS Balb/c mice provided complete protection after challenge with 100 PPFU of the CCHFV Turkey-Kelkit06 strain (Fig 6D). We previously showed that the cell culture based vaccine containing 20μg with three doses provided 80% protection in IFNAR^-/-^ mice challenged with 1000 PPFU [18], suggesting that complete protection ability of the cell culture based vaccine depends on the viral dose. At 96 hours post challenge, our titration analyses reveal that all vaccinated animals had significantly reduced viral burdens in the spleen, liver, and blood compared to mock-vaccinated mice (Fig 7A–7C). In our previous study, animals vaccinated with 20μg and 40μg doses of the cell culture based vaccine exhibited high levels of neutralizing antibodies and displayed 80% protection after the challenge. So, we claimed that neutralizing antibody responses are essential for the increased of protection of mice vaccinated with the cell culture based vaccine [18]. In contrast, the neutralization titers were approximately 2-fold higher in the cell culture based vaccinated mice than those mice receiving the mouse brain derived vaccine prior to challenge (Fig 3A–3C and Table 1), both the cell culture based and the mouse brain derived vaccinated mice provided full protection. These findings indicated that there was no direct correlation between high neutralization antibodies alone and protection, similar to what have been previously reported [13,15]. Previously passive transfer studies with monoclonal antibodies against Gn and Gc glycoproteins of CCHFV indicated that individual neutralizing and non-neutralizing antibodies alone can provide complete protection both before and after challenge in suckling mice [38]. In contrast, Golden et al. recently showed that the non-neutralizing mAb universally targeted the GP38 protein protected adult mice against lethal infection. They also found that anti-Gc neutralizing mAbs (mAb-11E7 and mAb-8A1) did not provide any protective benefit when given before or after virus challenge. [39]. Dowall et al. also found that the passive transfer of serum antibodies from MVA-GP vaccinated mice failed to protect naïve adult animals, despite neutralizing antibody activity [21]. A recent study demonstrated that immunization with transcriptionally competent virus like particles (tcVLPs) protected 40% of A129 mice despite the induction of strong neutralizing antibody titers [24]. Although we can not entirely exclude the neutralizing antibodies involved in protection increasing evidence shows that non-neutralizing antibodies play a significant role in protection in spite of how non-neutralizing antibodies have conferred to protection remains unclear.

In response to the recent outbreaks of CCHFV, there has been progress in improving our understanding of T cell responses to CCHFV. A modified vaccinia virus Ankara recombinant vaccine (MVAGP) for expressing the viral glycoproteins provided protection to require both cellular and humoral responses [15]. A recent report described a mouse model to CCHFV in which mice display severe clinical signs, high viral RNA loads, inflammatory cytokine responses, and organ pathology but most of infected mice recover from infection [40]. Authors concluded that recovery from infection correlated with long-lasting antibody and T-cell responses to CCHFV. A humanized mouse model of CCHFV demonstrated that T cells were activated following CCHFV infection and CD8+ T cells had elevated levels of perforin, a marker of cytolytic activity [12]. Currently, the only vaccine against CCHFV that is available for human use is the Bulgarian mouse brain derived vaccine reported that over a 22-year period the number of CCHF cases decreased four-fold following its introduction. Healty individuals vaccinated four times with the Bulgarian vaccine elicited T-cell response that were approximately ten-fold higher than those individuals receiving a single vaccination [30]. We previously developed the cell culture based vaccine against CCHFV but at this time we were not able to evaluate whether the vaccine was capable of activating the T-cell response [18]. In the present study, ELISpot analysis demonstrated a significantly increased IFN-γ response in all the vaccinated animals compared to mock-vaccinated animals (Fig 4). Notably, immunization with the cell culture based vaccine was capable of eliciting higher numbers of IFN-γ SFCs compared with the numbers observed for the mouse brain derived vaccinated mice (Fig 4). This findings suggest that induction of a cell-mediated immune response in Balb/c mice immunized three doses with two vaccine preparations might contribute protective immunity to CCHFV.

Here, we report that the IS mouse CCHFV model has been used to explore the immunogenicity and vaccine efficacy of the cell culture based and the mouse brain derived inactivated vaccines against CCHFV. Both vaccine preparations provided complete protection. However, the exact mechanism of protection remains unclear. The use of cell culture based inactivated vaccine against CCHFV provides several advantages over the use of inactivated mouse brain derived vaccine. Vero cells are the most widely accepted continous cell line by regulatory authorities for the manufacture of viral vaccines. Vero cells are devoid of neurological components which caused concerns with the inactivated mouse brain vaccine. The generation of virus in cell culture is much more cost effective than using mice.

## Supporting information

S1 Fig Antibody responses in vaccinated mice. Pooled sera from Balb/c mice, immunized with 20μg of the cell culture based vaccine (lane A, B and C) or 20μg of mouse brain derived vaccine (lane D, E and F) after two weeks of the last immunization were analysed by immunoblotting. Mock-vaccinated pooled sera (lane G) was a negative control. Each pool contained sera from 3–4 animals. Purified CCHFV samples were boiled and loaded onto a polyacrylamide gel. The samples were separated on 12% resolving and 5% stacking SDS-PAGE and the proteins were transferred onto a nitrocellulose membrane (Millipore, USA). After blocking with 5% skimmed milk, the membranes were probed with each pooled sera (1/1000) followed by a goat anti-mouse horseradish peroxidase (HRP)-conjugated antibody (1:1500 dilution, Invitrogen; USA). The membrane was exposed to an autoradiograph film (KODAK X-OMAT, Sigma Germany).
(TIF)
