# Supplementary figures and images for: Evaluation of the cell culture based and the mouse brain derived inactivated vaccines against Crimean-Congo hemorrhagic fever virus in transiently immune-suppressed (IS) mouse model
Source: PLoS Negl Trop Dis. 2020 Nov 23;14(11):e0008834. doi: 10.1371/journal.pntd.0008834 (PMC7721194; doi:10.1371/journal.pntd.0008834)

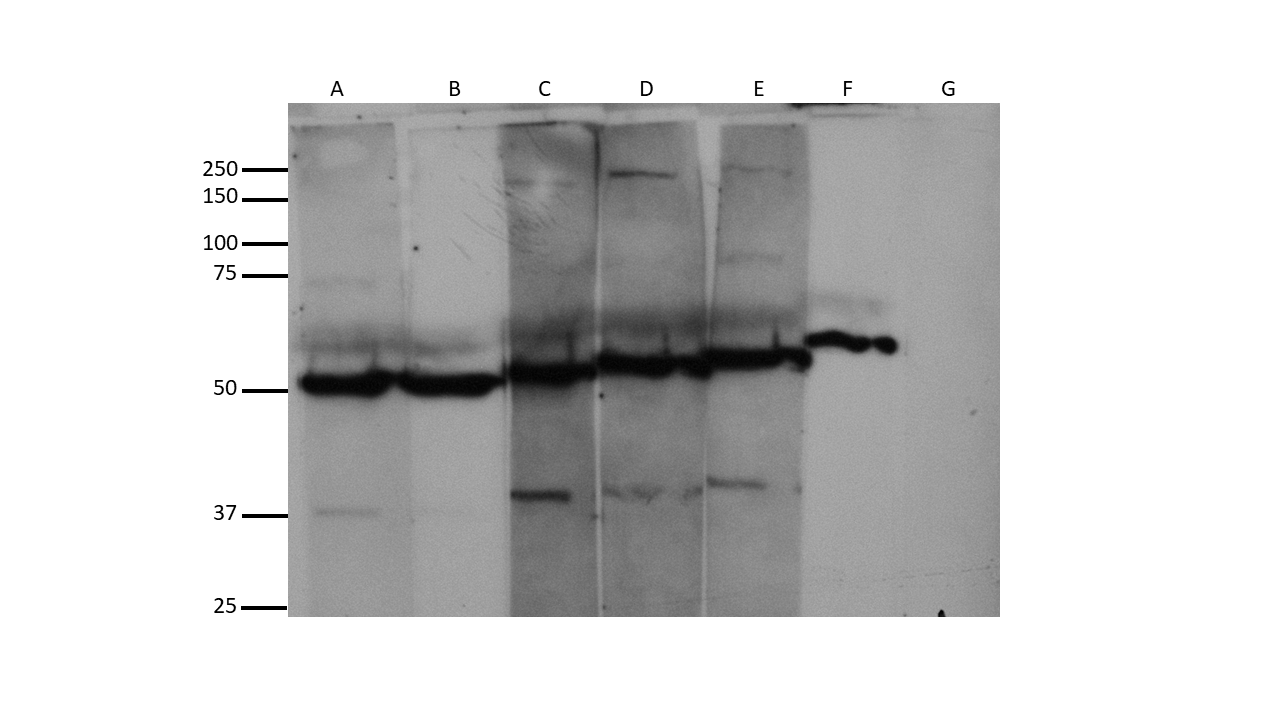

Supplement: S1 Fig — Pooled sera from Balb/c mice, immunized with 20μg of the cell culture based vaccine (lane A, B and C) or 20μg of mouse brain derived vaccine (lane D, E and F) after two weeks of the last immunization were analysed by immunoblotting. Mock-vaccinated pooled sera (lane G) was a negative control. Each pool contained sera from 3–4 animals. Purified CCHFV samples were boiled and loaded onto a polyacrylamide gel. The samples were separated on 12% resolving and 5% stacking SDS-PAGE and the proteins were transferred onto a nitrocellulose membrane (Millipore, USA). After blocking with 5% skimmed milk, the membranes were probed with each pooled sera (1/1000) followed by a goat anti-mouse horseradish peroxidase (HRP)-conjugated antibody (1:1500 dilution, Invitrogen; USA). The membrane was exposed to an autoradiograph film (KODAK X-OMAT, Sigma Germany). (TIF) [file pntd.0008834.s001.tif]
